# A review of data in medical request and the patient questionnaire for magnetic resonance evaluation of silicone breast implants

**DOI:** 10.1590/S1679-45082017AO4147

**Published:** 2017

**Authors:** Jaime Anger, Pablo Eduardo Elias, Paula de Camargo Moraes, Nelson Hamerschlak

**Affiliations:** 1 Hospital Israelita Albert Einstein, São Paulo, SP, Brazil; 2 Faculdade de Medicina do ABC, Santo André, SP, Brazil; 3 Hospital das Clinicas, Universidade de São Paulo, SP, Brazil

**Keywords:** Breast implantation, Silicones, Prostheses and implants, Magnetic resonance imaging, Surveys and questionnaires, Forms and records control, Implante mamário, Silicones, Próteses e implantes, Imagem por ressonância magnética, Inquéritos e questionários, Controle de formulários e registros

## Abstract

**Objective:**

To analyze the quality and quantity of data in the questionnaires and in request forms for magnetic resonance imaging.

**Methods:**

This retrospective study was conducted with data from 300 medical records. The research used the following data from the questionnaires: patient age, reason for the magnetic resonance imaging, reason for placing the breast implant, report of any signs or symptoms, time elapsed since surgery to place the current breast implant, replacement implant surgery, chemotherapy, and/or radiation therapy treatments. From the magnetic resonance imaging request forms, information about the breast implant, the implant placement surgery, patient clinical information and ordering physician specialty were verified.

**Results:**

The mean age of patients was 48.8 years, and the mean time elapsed since breast implant surgery was 5 years. A total of 60% of women in the sample were submitted to aesthetic surgery, while 23.7% were submitted to chemotherapy and/or radiation therapy. In the request forms, 23.7% of physicians added some piece of information about the patient, whereas 2.3% of them informed the type of implant and 5.2% informed about the surgery.

**Conclusion:**

The amount of information in the magnetic resonance imaging request forms is very limited, and this may hinder quality of radiological reports. Institutional and technological measures should be implemented to encourage the requesting physicians and radiologists to share information.

## RESUMO

**Objetivo:**

Analisar a qualidade e a quantidade de dados que constam nos questionários e nas requisições médicas de exame de ressonância magnética.

**Métodos:**

Estudo retrospectivo com 300 prontuários de pacientes. Dos questionários, foram utilizados os seguintes dados: idade, razão para a realização do exame, motivo para a colocação da prótese, referência a sinal ou sintoma, tempo decorrido desde a cirurgia de implante da prótese atual, se a cirurgia foi de troca da prótese, e se foi submetida à radioterapia ou à quimioterapia. Das requisições médicas do exame, foram utilizadas informações sobre prótese mamária, cirurgia de colocação do implante, dados clínico da paciente e especialidade do médico requisitante.

**Resultados:**

A média da idade das pacientes foi de 48,8 anos, e o tempo decorrido desde a colocação do implante foi de 5 anos, na média. Foram submetidas à cirurgia estética 60% das mulheres da amostra, e 23,7% tinham sido submetidas à quimioterapia e/ou radioterapia. Na requisição médica, 23,7% dos médicos inseriram algum dado, sendo que 2,3% informaram o tipo de implante e 5,2% sobre a cirurgia realizada.

**Conclusão:**

A quantidade de informações contidas nas requisições médicas foi baixa, o que pode comprometer a qualidade do laudo radiológico. Medidas institucionais e tecnológicas deveriam ser adotadas para estimular o intercâmbio de informações entre o médico solicitante e o médico radiologista.

## INTRODUCTION

Silicone breast implants are widely used to increase volume, improve the esthetic aspect of the breasts, breast reconstruction after cancer, correction of congenital breast abnormalities, and post-trauma or post-burn repair. The number of patients with implants has grown annually. In 2015, 1,488,992 breast augmentation surgeries with silicone implants were performed worldwide, 166,340 of them in Brazil. These data only refer to the procedures carried out by certified plastic surgeons.^(^ ^1^ ^)^

There are numerous manufacturers that produce several types of implants, which vary in form, external envelope surface, and content.^(^ ^2^ ^,^ ^3^ ^)^ Durability of these implants is not predictable, and the risk of loss of integrity increases over time from its assembly, and especially, depending on the quality of its production.^(^ ^4^ ^)^

Many complications may occur in patients with breast implants, such as rupture of the envelope and silicone leakage, dislocation of the implant, increased consistency of the surrounding tissues, and appearance of fluid collections in the region.^(^ ^5^ ^)^ In addition to characteristics of the implant used, surgical technique used, cause of the operation, and patient's clinical condition are also determining factors of complications.

One of the most frequent and most studied complications is the prosthetic loss of integrity. This implies a new operation for its removal or replacement. There may be rupture due to a technical error during the surgery or, more frequently, due to manufacturing failure of the implant. In 2010, the French agency *Agence Nationale de Securité du Médicament et des Produit de Santé* suspended the use of gelatinous silicone breast implants produced by *Poly Implant Prothèse* (PIP), due to the use in its manufacture of non-approved material that could cause loss of integrity of the implants, and the removal of these implants from the market was recommended.^(^ ^6^ ^,^ ^7^ ^)^

Rupture of the envelope is asymptomatic in up to 50% of patients, and the presence of fluid collections is difficult to identify by physical examination.^(^ ^8^ ^)^ The difficulty in reaching a clinical diagnosis of most complications leads to the need to use imaging diagnostic tests. Among the modalities of tests available, mammogram and ultrasonogram are not ideal to evaluate implants and their complications; magnetic resonance is considered the reference test for this.^(^ ^9^ ^–^ ^12^ ^)^ In 2006, the Food and Drug Administration (FDA) recommended implant follow-up by magnetic resonance three years after insertion and a repeat test every two years.^(^ ^6^ ^)^

When preparing a precise radiological report it's important that the radiologist is given access to the patient's clinical and surgical data. However, in most cases these information aren't available in the image request made by the attending physician and are only obtained with the patient at the day of the exam.

## OBJECTIVE

To analyze quality and quantity of data in medical request forms for magnetic resonance imaging tests of breasts in patients with silicone breast implants.

## METHODS

This is a retrospective study where 300 medical records from the Radiology Department at the *Hospital Israelita Albert Einstein* were analyzed. The patient profile for this study was composed of female patients with silicone breast implants that went thru a magnetic resonance between December 12th 2012 and August 17th 2013.

Data were collected from the questionnaires handed out to patients right before the test, filled in by a member of the nursing team with the answers given by patients. The data in the medical requests for these tests were also analyzed. A total of 300 medical records were studied after exclusion of 46 that did not contain a medical request or the questionnaire available.

From the questionnaires, the following data were utilized: age, reason for the test, reason for implant placement, reference to signs or symptoms specifying the location and laterality, time passed since the operation to place the current implant, if the surgery was to substitute the implant, and if the patient was submitted to radiation therapy or chemotherapy.

Of the medical requests for the test, we utilized information about the breast implant, the operation for breast implant substitution, clinical condition in reference to some signal or symptom, and the specialty of the ordering physician.

### Data analysis

Categorical variables were described by means of absolute and relative frequencies, and quantitative variables, through summary measures such as mean and standard deviation, or median and interquartile range (IQR), besides the minimum and maximum values.

The distribution of the quantitative variables was studied by histograms and boxplots, and the Shapiro-Wilk normality test was used. The Mann-Whitney tests were applied in the comparison between the requests with and with no data about time of implant placement.

Associations between the presence of data on the medical requests and the variables of interest were analyzed by χ^2^. The analyses were performed using the Statistical Package of Social Science (SPSS), considering a significance level of 5%.

This study was approved by the Research Ethics Committee, under number 352.947, CAAE: 18452813.4.0000.0071. The patients' medical records belong to *Hospital Israelita Albert Einstein* , and are exempt from the Informed Consent Form (ICF).

## RESULTS

The data analysis contained in the prior questionnaire showed that the age of women at the time of the test varied between 17 and 82 years, with a mean age of 45.8 years (standard deviation of 11.6 years) ( Table 1 ).

**Table 1:** Descriptive analysis of the data contained in questionnaires answered by patients

Age, years		
	Mean (SD)	45.8 (11.6)
	Minimum-maximum	17-82
Primary reason for the test, n (%)		
	Assessment after breast cancer	93 (31.0)
	Assessment after esthetic surgery	180 (60.0)
	Breast nodule	27 (9.0)
Clinical complaint or reported physical abnormality, n (%)		
	No report	206 (68.7)
	Yes	94 (31.3)
The last surgery was a prosthetic replacement, n (%)		
	No report	241 (80.3)
	Yes	59 (19.7)
How many years ago was the implant placed?		
	Median (IQR)	5.0 (2.0-9.0)
	Minimum-maximum	1-30
	Patients, n	264
Was patient submitted to radiation therapy? n (%)		
	No report	261 (87.0)
	Yes	39 (13.0)
Was patient submitted to chemotherapy? n (%)		
	No report	259 (86.3)
	Yes	41 (13.7)
Radiation therapy and chemotherapy, n (%)		
	No report	246 (82.0)
	Yes	54 (18.0)

SD: standard deviation;

IQR: interquartile range.

Most tests were performed on patients who had been submitted to prosthetic implant surgery due to esthetic reasons (60.0%); 30% on patients who had been submitted to breast reconstruction for primary breast cancer.

In the questionnaire, 31.3% of patients reported presenting with some physical abnormality or breast symptom.

Time elapsed between placement of implant and test request varied between one and 30 years, with a median of five years (IQR: 2 to 9 years); moreover, 19.7% of patients reported they had already had the implant replaced at least once.

As to treatment, 13.0% of patients underwent radiation therapy, 13.7% chemotherapy, and 18.0% both radiation therapy and chemotherapy.

In 23.7% of requests, the physicians provided at least one piece of information on the implant, clinical or surgical data. In 19.0%, there was only one piece of information, 3.0% contained two pieces of information, and only 1.7% provided three pieces of information. Of the information given, 2.3% were about the implant, 5.3% about the surgery to place the current implant, and 22.3% about the presence of a sign or clinical symptom ( Table 2 ).

**Table 2:** Descriptive analysis of the data contained in the medical requests

Data on the medical requests		n (%)
Specialty of the ordering physician		
	Gynecology or Mastology	175 (58.3)
	Plastic Surgery	59 (19.7)
	Oncology or Radiation Therapy	12 (4.0)
	Internal Medicine (any clinical specialty)	29 (9.7)
	Other specialties	8 (2.7)
	Not reported	17 (5.7)
Are there data about the implant in the request forms?		
	No	293 (97.7)
	Yes	7 (2.3)
Are there clinical data on the request forms?		
	No	233 (77.7)
	Yes	67 (22.3)
Are there surgical data on the request forms?		
	No	284 (94.7)
	Yes	16 (5.3)
Any data provided by the physician		
	No	229 (76.3)
	Yes	71 (23.7)

Most test requests (58.3%) were made by Gynecologits, followed by Plastic Surgeons (19.7%); the other specialties summed up 22% of requests ( Table 2 ). Considering the two most frequent specialties (Gynecology and Plastic Surgery) there was evidence of an association between the specialty and the presence of data on the requests (p<0.001). The results suggest that plastic surgeons provide more information than gynecologists (49.2% of plastic surgeons *versus* 18.9% of gynecologists).

We noted evidence of an association between presence of any datum provided by the physician and the primary reason for the test (p=0.006). Yet, the proportion of data provided by physicians on requests due to cancer or esthetics was greater than on the request forms for breast nodules.

There was evidence of differences between requests with and with no data regarding time elapsed since implant placement (p=0.026). The median time of placement of implant in requests with information (median 7; IQR: 3-11) was greater than in those without information (median 5; IQR: 2-8).

There was no evidence of an association between any data provided by physicians and the following variables: the patient having undergone radiation therapy or chemotherapy (p=0.938), surgery for prosthetic substitution (p=0.311), and the presence of a clinical complaint (p=0.272).

## DISCUSSION

The request of an imaging test is usually made by a physician after clinical evaluation of the patient. This reality is not different in patients with breast implants; actually, in the specific case of these patients, there was an increase in orders for imaging methods, since they are superior to physical examination in detecting complications related to the implants.

In this research, it is relevant to verify there is little information in the request forms. Only 2.3% contained data on the type of implant. Even after repeated scandals involving failures in implants of some manufacturers in recent years, most request forms had no information about the brand of the implant the patient had. The same is true in reference to the lack of information on implant shape. In 2017, an imaging study demonstrated that it is possible to identify if the asymmetric implants changed position and axis.^(^ ^13^ ^)^

Only 5.3% of requests contained information about the surgery to place the current implant. In the questionnaires, 19.7% of patients reported they had undergone a prosthetic substitution. The cause of replacement and the surgical technique used, as well as the presence of anatomical abnormalities, are relevant when preparing the imaging test report. When surgery is performed to replace a ruptured implant, if the leaked silicone is not removed, its presence in tissues can modify the interpretation of the image.^(^ ^14^ ^)^

When answering the questionnaire before the test, 31.3% of patients reported clinical complaints, and 31% reported they had already been submitted to chemotherapy or radiation therapy. Nevertheless, only 22.3% of medical requests contained any clinical data. There is an evident *deficit* of information on the requests, considering that one third of the tests were requested for follow-up of breast cancer patients.

Most request forms were made by gynecologists (58.3%). Although in smaller number (19.7%), the requests made by plastic surgeons had more information about the implant. Plastic surgeons can provide more information since they are responsible for placing the implants.

Several studies demonstrated that there are numerous factors associated with errors and discrepancies in imaging test reports,^(^ ^15^ ^,^ ^16^ ^)^ and the rates range from 3 to 11%. However, these can be minimized, if radiologists have access to a greater quantity of clinical and laboratorial data about patients.^(^ ^17^ ^)^ One way to reach this objective is by sharing information with the ordering physician.^(^ ^18^ ^)^

Since it is clear there is few data provided in our study, some actions could be implemented, such as awareness that ancillary tests are part of the diagnosis process, and that the ordering physicians are responsible for what was indicated or what they have participated in. Hospitals, professional societies and regulatory agencies, have been concerned with providing technical conditions and humanization practices for radiologists. Therefore, we consider that these organizations should also promote means of making others aware and have active protocols, to encourage physicians to share more information with radiologists, including as much data as possible in their test requests, reducing the possibility of undesirable events.

## CONCLUSION

The amount of information in the medical request forms for magnetic resonance imaging of the breast in patients with breast implant was low, and this could compromise quality of the radiological report. Guidance about the importance of clinical information in request forms, or development of standardized medical requests should be adopted to foster greater exchange of information among ordering physicians and radiologists.
